# Researches upon the Venom of the Rattlesnake

**Published:** 1861-07

**Authors:** 


ART. V.—*Researches upon the Venom of the Rattlesnake: with an Investigation of the Anatomy and Physiology of the Organs concerned.* By S. WEIR MITCHELL, M.D., Lecturer on Physiology in the Philadelphia Medical Association. Published by the Smithsonian Institution: Washington City, January, 1861. 4to., pp. 145.

THE most pestilent member of a repulsive family, the very type of hideous and untamable malice, loathsome in repose, and frightful in activity, the rattlesnake can hardly be looked upon as an agreeable object even by a scientific eye. It is, therefore, with a feeling very much like gratitude that we examine this record of nearly two years of laborious and hazardous investigation in this branch of toxicology. Dr. Mitchell has conferred a benefit not only upon science, but upon all who may at any time be exposed to rattlesnake poisoning, and thus has exercised one of those functions which go to constitute the patent of nobility of our profession. In the present notice we shall, for obvious reasons, abstain from criticism, for which, indeed, we see no opening, and assume the humbler task of laying before our readers an analysis of this excellent monograph.

Dr. Mitchell's researches have been directed to the clearing up of points in connection with the anatomy and physiology of the venom apparatus in the rattlesnake, to the chemistry of the subject, and to certain phenomena which cannot be studied to advantage in clinical cases, uncommon as they happily are, and distributed, as they must necessarily be, among a number of unequally competent observers. He has pursued these latter inquiries by means of experiments upon the lower animals; investigating the mode of action of the poison, its effects when administered in different ways, and the circumstances which hinder or promote its activity. Of these experiments a most interesting account is given. Another chapter is devoted to the subject of crotalus poisoning in man, and its treatment,* with a tabular summary of the cases hitherto reported.

It would be useless for us to attempt to give our readers an exact idea of the anatomical details of the poison apparatus, without the aid of illustrations; but there are some general facts which may be mentioned, if merely on account of the interest they possess.

Most persons are probably aware that the head of the rattlesnake, like that of other snakes, is so constructed as to allow of a good deal of motion between the several bones; and that the poison fangs, when not in use, are laid back along each side of the roof of the mouth, inclosed in a sheath of the mucous membrane. Each fang is planted firmly by its upper end in the upper maxillary bone, which is jointed to the lachrymal bone above, to which, at the upper end of its posterior surface, is attached the anterior end of the ecto-pterygoid bone. This latter bone is long and slender, and, when drawn forward by the spheno-pterygoid muscle, must push the upper maxillary bone forward also, causing it to rock upon the lachrymal bone, and this again upon the frontal. It is astonishing how widely this whole arrangement enables the snake to separate the jaws.

* In a former issue of this Review (March, 1861) may be found a paper by Dr. Mitchell, on "The Treatment of Rattlesnake Bites," in which the therapeutical aspects of crotalus poisoning are very fully discussed.

Of the muscles concerned, the anterior temporal is the most important, since it not only aids in the closure of the jaws, but compresses the poison gland. This gland, closely resembling in structure the typical salivary gland, lies just below and behind the eye, and is about four times as long as it is wide. Its capsule, formed of strong, white, fibrous tissue, gives off processes which constitute suspensory ligaments. Its duct, when the head is at rest, is nearly of an ∽ shape, and terminates at the anterior part of the fang, where a fissure becomes continuous with it. A well-marked sphincter enables the snake to control the flow of venom completely. When the fang is raised, the curves in the duct are obliterated, except just where it turns downward, at the sphincter. Dr. Mitchell quotes Owen's description of the fang:—

"To give an idea of the structure of this tooth, we may suppose a simple, slender tooth, like that of a boa-constrictor, to be flattened, and its edges then bent toward each other, and soldered together so as to form a tube, open at both ends, and inclosing the end of the poison duct. The duct which conveys the poison, although it runs through the centre of the tooth, is really on the outside of the tooth. The bending of the dentine beyond it begins a little beyond the base of the tooth, where the poison duct rests in a slight groove or longitudinal indentation, on the convex side of the fang. As it proceeds, it sinks deeper into the substance of the tooth, and the sides of the groove meet and coalesce, so that the trace of the inflected fold ceases, in some species, to be perceptible to the naked eye, and the fang appears, as it is commonly described to be, perforated by the duct of the poison gland."

Behind the root of the fang, in the maxillary bone, are cavities of reserve, in which fresh fangs lie ready to take the place of the fully developed one, when this is either shed or violently detached. This curious arrangement has been accurately studied by Dr. Christopher Johnston, of Baltimore, whose account of it is given by Dr. Mitchell; but our limits forbid our quoting it here.

The mechanism of the bite would seem to be somewhat complicated. According to Dr. Mitchell, the force of the forward motion of the head in striking is not very great, nor is its range more than one-third or one-half the length of the snake. The jaws are widely separated, and the head is thrown back so as to cause the point of the fang to project almost directly forward; but the elevation of each fang is a separate act of the animal, either, neither, or both being brought into play at will. When the blow is given, and the fang has entered the flesh, it is so acted upon through the palate bone, by certain muscles, as to drive it in more deeply, the whole head being meanwhile fixed by yet other muscles. Now the jaws are brought into antagonism, mainly by the temporal muscles, and this of course forces the fang still further in; but the more important element of the action is the squeezing of the gland, and the consequent ejection of the venom into the wound.

Should there be any entanglement of either jaw, the snake may shake its head from side to side in trying to free it, a movement which has been incorrectly thought expressive of rage. If otherwise, the muscles being relaxed and the motions reversed, the fangs are withdrawn at once. Dr. Mitchell has noticed that, when the snake is confined at the time of biting, the fangs often diverge from one another; he thinks that this is the case also when the snake is free, and that its object is to protect the lower jaw from injury.

We should be glad to mention some points of minor importance, but of great interest, relating to the provisions for restraining the flow of the poison at times when it is not desired, and to the various circumstances which may render the bite ineffectual. We must, however, be content with this allusion. A knowledge of these latter, it may be remarked, may account for the supposed efficacy of some antidotes for the venom; the fact being that in some of the cases treated the injury was either imperfectly inflicted, or not at all.

The capacity of the gland is not more than a few drops. In snakes which have been long inactive the poison accumulates. Dr. Mitchell saw one, which probably had not used its fangs for several weeks, eject fifteen drops. As to the physical characters of the venom, it is a yellowish or green liquid, glutinous, free from taste or smell, distinctly acid, with a specific gravity somewhere above 1030. When kept too long, it becomes very offensive, and vibriones and confervoid growths become developed in it; but its virulence remains. Neither in the recent nor the decomposed state does it exhibit any important microscopical characters.

Chemically examined, this poison is found to contain, besides coloring matter and an undetermined substance, both soluble in alcohol, a trace of fatty matter, chlorides and phosphates, and two albuminoid matters. One of these latter is coagulable at 212° F. The other, *crotaline*, not coagulable at 212°, is of neutral reaction, freely soluble in water, and nitrogenous in its nature. Dr. Mitchell is of opinion that the analogy, which might be on anatomical grounds supposed to exist between the venom and its gland and the saliva of man and his salivary glands, has no reality.

Changes of temperature have no effect whatever upon the activity of rattlesnake poison; nor is it impaired by mixture with alcohol, strong acids or alkalies, chlorine or iodine.

It has been asserted by some observers that this poison has a deleterious influence upon vegetable organisms. Dr. Mitchell, however, was unable to confirm the statement, although he found that seeds, placed in otherwise favorable circumstances for germination, failed to undergo that change if in contact with the venom.

Of cold-blooded animals, the frog affords the best opportunity for toxicological experimentation. Upon it rattlesnake poison produces two sets of symptoms, acute and chronic, or primary and secondary; but these are less distinct than in the case of warm-blooded animals. Occasional muscular convulsions, and loss of voluntary power, precede death; the part bitten is apt to be infiltrated with blood, and the tissues just around the bite are often disorganized. Sometimes the lungs are either dotted with ecchymosis or gorged with blood; the blood in the heart may or may not be coagulated. When dried venom is inserted beneath the skin, the local effects are much less pronounced than when a snake inflicts its bite.

The prevalent idea that a snake may poison itself is confirmed by Dr. Mitchell's observations, in opposition to the view entertained by Fontana with regard to the viper. We must, however, remark that we do not understand why a fatal result never ensued when, as Dr. Mitchell says was often the case, the venom was brought freely in contact with abraded surfaces in the mouths of snakes.

Birds, rabbits, guinea-pigs, and dogs were the warm-blooded animals employed in the experiments referred to in this paper. The symptoms varied in different cases, as it might *a priori* be supposed they would; but the longer life lasted in any instance, unless the animal ultimately recovered, the more serious were the internal lesions observed. Extravasation of blood and softening of the tissues in the neighborhood of the bite were almost invariably caused; the nerve centres were more or less injected, the heart distended and flabby, the lungs sometimes gorged, and the intestines occasionally ecchymosed. In one case, that of a small dog, the kidneys "were full of blood, and presented the appearance of acute congestion. On further inspection, a long thin clot was found in the left ureter, and bloody urine in the bladder below."

A very interesting field of inquiry is presented in regard to the action of the venom upon the different tissues and fluids. Dr. Mitchell does not think that it can pass through the unbroken mucous coat of the alimentary canal, but is inclined to believe that of the pulmonary tract a less effective barrier to it. Blood effused in a wound, in contact with the venom, is hindered from coagulating, unless the latter is in very small relative quantity. To muscular tissue the poison is a direct and active irritant, ultimately, however, causing disintegration.

With regard to the effect of the poison on the heart, an important but difficult subject of inquiry, Dr. Mitchell offers some very interesting statements. In frogs the organ seemed to be enfeebled; but this circumstance was clearly distinct from the cause of death. In warm-blooded animals the phenomena produced are quite different from those which ensue upon the action of certain poisons, which are known to owe their effects to their paralyzing influence upon the heart. We should be glad to quote freely our author's account of his researches in reference to this matter, but our limits forbid; and we could do him no justice did we attempt to condense it.

No effect whatever is produced by rattlesnake poison on the capillaries, on the intestinal walls, or on the cilia of epithelial surfaces.

Upon the nerve trunks direct contact of the venom had no visible influence. Careful experiments seemed to warrant the conclusion that the central portion of the nervous system was primarily acted upon, in the way of paralysis.

The temperature of the body always falls in this as in other forms of poisoning, and hence may be derived a therapeutical hint as to the importance of keeping it up by artificial means.

Blood, although it coagulates as usual when mixed with rattlesnake poison, does not remain so; the clots become softened, or even wholly redissolved, especially if the quantity of venom is large and the temperature high. This fact goes to explain the fluid state of the blood in cases of chronic poisoning, where the period of contact has been, of course, prolonged. Dr. Mitchell considers the agency of the venom as exerted not in preventing the development of new fibrin, but upon that already formed, as in the case of other septic poisons. The blood-corpuscles are unaffected; in the guinea-pig their contents crystallize, as they would after death from any other cause.

With regard to the causes of death by rattlesnake poisoning, we may quote Dr. Mitchell's own words :—

"Perhaps scarcely one intelligent medical reader will have followed me thus far without arriving at the conclusion that the venom of the crotalus, like that of other snakes, is a septic or putrefacient poison of astounding energy. This very obvious view has long been held by toxicologists, and the cases and experiments of this paper assuredly do not weaken it.

"The rapid decomposition of the blood and of the tissues locally acted on by the venom leaves no doubt upon the matter, and makes it apparent that an incipient putrefaction of this nature may so affect the blood as to destroy its power to clot, and perhaps, also, to nourish the tissues through which it is urged.

"The alterations thus brought about are probably the results of a continued fermentative change, which, begun by a small amount of poison, is gradually made to involve in fatal change the whole mass of the circulating fluids. Like all fermentations, however, the rapidity depends on temperature and on the amount of the primary ferment. In one instance, a dog, struck by eight snakes, died in eighteen minutes, and exhibited an uncoagulable blood. I am aware of no other case of loss of coagulating power so rapid. It was rendered thus by the number of localities from which the ferment attacked the system. On the other hand, a frog, a small animal, receives the same dose of venom as would have entered the tissues of a larger animal, yet it resists the poison most remarkably, by virtue of its powers as a cold-blooded creature, existing at the temperature of the atmosphere itself.

"Admitting, then, that the changes effected in the blood may be sufficient to account for the fatal results in chronic cases of poisoning by crotalus, are we justified in referring to similar causes the sudden deaths which sometimes take place in small, or even larger animals in whose tissues or fluids we can detect no change whatsoever?

"In the present state of this inquiry, the question scarcely admits of a positive answer. It is clear that in acute cases the symptoms of depression are most marked, and the heart and nerve centres are suddenly and fearfully enfeebled, so that their irritability is lessened, and is finally lost earlier than occurs in other forms of death. If, now, we knew of no other property of the poison than this one we would properly pause here, and regard the venom as having a specific influence on the heart and on the nervous irritability of some part of the cerebro-spinal centres, such as characterizes certain of the better-known poisons, such as corroval, woorara, upas, opium, aconite, etc. Since, however, we are aware that serpent venom, after remaining for a time in the body, has a specific power of attacking at least one element of the blood, and through its degradation, perhaps, of affecting the whole circulating fluid, we are naturally inclined to ask whether this power may not also be invoked to explain even the *ultimate* nature of the *sudden* cases of death from the venom. If, for instance, a pigeon is struck by a snake, and dies in thirty seconds, its tissues normal in appearance, and its blood unaltered, have we any logical right to infer that the blood may have been inappreciably, but fatally, altered, so as to be unable to sustain the life of the tissues which it feeds? Is it this possible, but imperceptible, change in the blood which acts to produce those losses of irritability in the nerve centres which we have been led to regard as the proximate cause of early and rapid death? If such be the case, then *the suddenness of the general change* in the blood must account for the failure of life, because it can be shown that animals whose blood is considerably altered may live for some time, or even survive to renew and refibrinate their vital fluids.

"*The cause of death in chronic or secondary poisoning* may, with propriety, then, be referred to the incipient putrefactive changes which affect the blood, as well as to the continued influence of the agencies which first act to depress the heart's action, and destroy nerve function.

"*The cause of death in acute cases*, when the result is so sudden that no change is perceptible in the blood in the vessels, is amply explained in the preceding pages; but while we are able to state where death begins, and in what order the functions succumb, we are still far from knowing why, or precisely how, this or that structure is affected. The proximate causes are open to experimental study, the ultimate reason, as we have seen, (page 95,) is as yet unknown.

"Summing up, then, what we have learned of the acute form of poisoning, we may feel justified in concluding: 1st. That the heart becomes enfeebled shortly after the bite. This is due to direct influence of the venom on this organ, and not to the precedent loss of the respiratory function. Notwithstanding the diminution of cardiac power, the heart is usually in motion after the lungs cease to act, and its tissues remain for a time locally irritable. The paralysis of the heart is, therefore, not so complete as it is under the influence of upas or corroval.

"2d. That in warm-blooded animals, artificial respiration lengthens the life of the heart, but does not sustain it so long as when the animal has died by woorara, or decapitation.

"3d. That in the frog, the heart-acts continue after respiration has ceased, and sometimes survive until the sensory nerves and the nerve centres are dead, the motor nerves alone remaining irritable.

"4th. That in warm-blooded animals respiration ceases, owing to paralysis of the nerve centres.

"5th. That the sensory nerves and the centres of nerve power in the medulla spinalis and medulla oblongata lose their vitality before the efferent or motor nerves become affected.

"6th. That the muscular system retains its irritability in the cold-blooded animals, acutely poisoned, for a considerable time after death.

"7th. That the first effect of the venom being to depress the vital energy of the heart and nerve centres, a resort to stimulants is clearly indicated, as the only rational mode of early constitutional treatment."

From these conclusions, as well as from a few remarks which succeed them as to the analogy between rattlesnake poisoning and certain diseases, it may be seen how obscure and difficult this whole subject is, and moreover how important it is to bring together the results of our researches in various paths, the light thus obtained being not unfrequently far more valuable than might have been expected, and none the less so for its indirectness.

Dr. Mitchell has been led by his investigations to the opinion that—

"Whatever may be the degree of virulence in the poison of different venomous snakes, its mode of affecting the system varies but little, whether the bite be inflicted by the viper, the copperhead, the rattlesnake, or the dreaded, but not more deadly, cobra. Thus, in each case, we have the local poisoning, the constitutional malady, and the possibility of inexplicably rapid death on the one hand, and of a strange zymotic disease upon the other. There may yet remain some room for doubt as to whether the apparent difference in the activity of the venom from various serpents is not due to the quantities formed or stored up in each case, and to unobserved peculiarities in the structure and form of the poison apparatus. However this may be, it is quite certain that two cases of rattlesnake poisoning may sometimes differ as much as either one of them will from a case of moccasin or cobra bite."

We have not space to examine at any length the concluding chapter of this essay, on rattlesnake poisoning in man. It contains a tabulated statement of sixteen reported cases, four only of which were fatal. A very interesting practical fact mentioned is, that the gravest symptoms often become suddenly dissipated, so as to give rise to special but groundless confidence in any remedies which may have been made use of. We have, however, already remarked that the readers of this Review may find Dr. Mitchell's views in regard to the treatment of crotalus poisoning set forth at length in a previous number, so that it would be needless for us to repeat them here.

Appended to this paper is an enumeration of the genera and species of rattlesnakes, with synonymy and references, by Mr. E. D. Cope, an experienced herpetologist; and Dr. Mitchell has given a bibliographical table, which has evidently been carefully prepared. We must confess our surprise at the extent of the literature of this branch of toxicology.

We are aware that we have not, in the foregoing notice, done full justice to Dr. Mitchell's most valuable essay. This, however, has been necessarily the case, from the number of experimental details contained in it, and from the condensation which marks its style. But we have endeavored to set forth its leading features, and to give an idea of its character. In conclusion, we would congratulate the friends of science in America upon the existence of the Smithsonian Institution, supplying as it does the want of literary patronage on the part of Government. But for its fostering care, such papers as that of Dr. Mitchell would in many cases be lost to the world for want of the proper auspices for publication.

